# Coronary microvascular dysfunction and atrial reservoir function

**DOI:** 10.1186/s12947-024-00324-0

**Published:** 2024-05-14

**Authors:** Julien La Mela, Laurent Riou, Gilles Barone-Rochette

**Affiliations:** 1 Department of Cardiology, University Hospital, Grenoble, 38000 France; 2 University Grenoble Alpes, INSERM, CHU Grenoble Alpes, LRB, Grenoble, 38000 France; 3 French Clinical Research Infrastructure Network, Paris, 75018 France; 4 Clinique Universitaire de Cardiologie, Pôle Thorax et Vx, CHU de Grenoble, 38043 Grenoble cedex 09, France

**Keywords:** Coronary microvascular dysfunction, Left atrial strain reservoir

## Abstract

**Background:**

Coronary microvascular dysfunction (CMD) refers to structural and functional abnormalities of the coronary microcirculation, which may be diagnosed using invasive coronary physiology. CMD is responsible for impaired diastolic cardiac function. It has recently been suggested that left atrial strain (LASr) represents a highly sensitive tool for detecting cardiac diastolic function abnormalities. Accordingly, the aim of this study was to investigate the relationship between CMD and LASr.

**Methods:**

Consecutively enrolled patients with non-obstructed coronary arteries (NOCA) underwent CMD and LASr evaluation by invasive thermodilution and noninvasive echocardiography, respectively.

**Results:**

Forty-two (42) patients were included, out of which 26 presented with CMD. There were no significant differences between CMD-positive and negative patients in terms of clinical and echocardiographic characteristics. LASr was significantly reduced in patients with CMD (24.6% ± 6.1 vs. 30.3 ± 7.8%, *p* = 0.01). A moderate correlation was observed between coronary flow reserve and LAsr (*r* = 0.47, *p* = 0.002). A multivariate logistic regression analysis demonstrated that CMD was independently associated with LASr (OR = 0.88, 95%CI 0.78–0.99.135, p = 0.04). A LASr cut-off of 25.5% enabled an optimal classification of patients with or without CMD.

**Conclusion:**

Patients with NOCA and CMD had a significantly reduced LASr compared with patients without CMD, suggesting the early impairment of diastolic function in these patients.

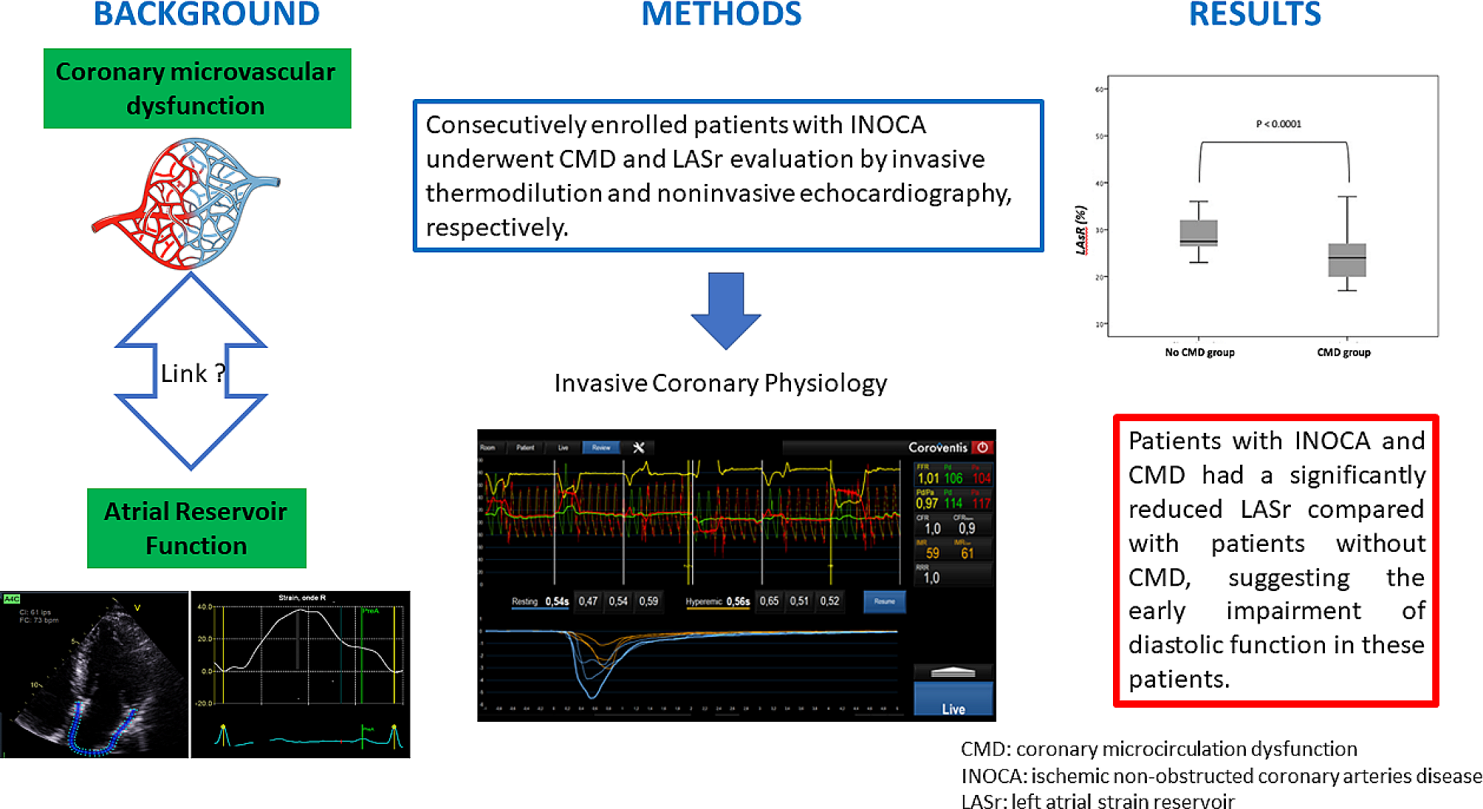

## Introduction

Coronary microvascular dysfunction (CMD) corresponds to structural and/or functional damage to small (< 400 μm) coronary vessels. Patients with CMD experience a reduction in coronary flow reserve, leading to abnormal flow at rest or an altered coronary response to stress. Recent studies reported that 47–60% of patients with typical angina-like symptoms and no significant epicardial lesions according to coronary angiography have CMD [1, 2]. The diagnosis of CMD has been standardized based on clinical criteria and invasive and/or non-invasive imaging tests [3, 4]. Studies have shown that CMD is associated with an increased incidence of cardiovascular events including myocardial infarction, stroke and hospitalization for heart failure [1]. The role of CMD in heart failure with preserved ejection fraction (HFpEF) has also recently been described by a number of studies, including its role in the development of diastolic disorders characterizing this pathology [5–7].

Technological advances in echocardiography allow the analysis of myocardial contraction and deformation through left atrial strain (LAS). LAS allows the assessment of all three aspects of left atrial function, namely reservoir (LASr), conduit and pump [8–10]. The LASr technique is robust, easy to use, has low intra- and inter-observer variation [11, 12], and is used in a variety of cardiac pathologies [10–12]. LASr is now proposed as part of the algorithm used for the assessment of left ventricular filling pressures [8, 13]. It is also considered as a prognostic biomarker, especially in atrial fibrillation where it indicates the probability of success of an external electric shock, and it is a predictive factor for stroke [12, 14–16].

The aim of this study was to analyze the relationship between LASr and CMD as assessed by invasive coronary physiology.

## Methods

### Study population

**Fig. 1:**
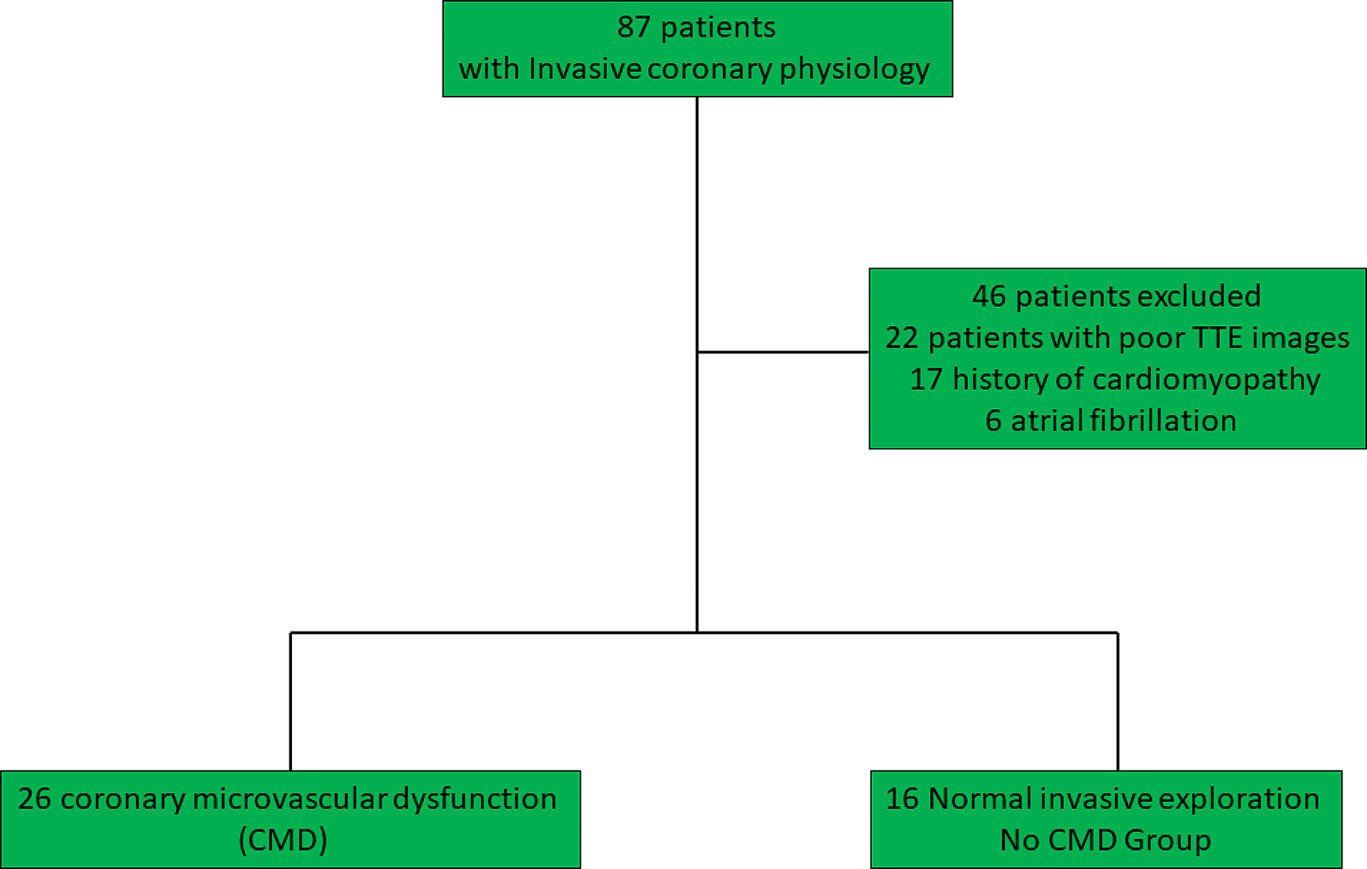
Flow Chart. TTE: Trans-Thoracic echocardiography

The present prospective and single-centered study recruited consecutive patients referred for coronary angiography upon clinically appropriate indications (ClinicalTrials.gov identifier: NCT04560829). The study included men and women with chest pain suggestive of angina and/or positive ischemic test and non-obstructed coronary arteries (NOCA), who underwent invasive coronary physiology studies between September 2020 to June 2023. Exclusion criteria were (a) contraindications to adenosine (asthma, second or third-degree AV block or sick sinus syndrome without a pacemaker, methyl xanthenes medication within the last 12 h prior to the test, and known hypersensitivity to adenosine); (b) previous percutaneous coronary intervention or coronary artery bypass graft surgery; (c) previous myocardial infarction; (d) pregnancy; (e) low ejection fraction < 50%; (f) known cardiomyopathy or severe valvular disease; (g) coronary stenosis > 50% or fractional flow reserve (FFR) < 0.8. A total of 42 patients satisfied the inclusion criteria (Fig. 1) and constituted the final study population. The institutional review board approval was obtained as per current regulations. The sponsor of the trial was our University Hospital. The study protocol was in accordance with the Declaration of Helsinki.

### Invasive coronary physiology

Selective conventional invasive coronary angiography (ICA) was performed using standard techniques using the Judkins technique and a radial approach with 6-French catheters (Philips Allura Xper FD10, Philips Healthcare). After selection of patients with no stenosis > 50% by angiography, left ventriculography, as ultrasound was performed after the invasive examination, was performed to ensure that there is no alteration of the left ejection fraction, and that there are no abnormalities in filling (N ≤ 10 mmHg) in accordance of the expert consensus [4]. This process it is important because Many patients with HFpEF would fulfil these criteria: dyspnoea, no obstructive CAD and impaired CFR. FFR, CFR and IMR were determined mainly on the left anterior descending artery (LAD). The IMR was assessed using a coronary pressure wire (PressureWire X, Abbott, IL, USA) at maximal hyperemia induced by intravenous adenosine (140 µg/kg/min). The coronary pressure wire was flushed. Following calibration to zero pressure, the coronary pressure wire was equalized to the guide catheter pressure with the sensor positioned at the ostium of the coronary artery. The pressure sensor was then positioned at the distal segment of a target vessel, and intracoronary nitrate (100 or 200 mg) was administered before each measurement. Resting mean transit time (Tmn) was obtained in triplicate by rapid intracoronary manual administrations of a 3 mL bolus of saline at room temperature. Hyperemia was induced by intravenous adenosine (140 µg/kg/min) during at least 3 min via a peripheral or central vein. Hyperemic mean aortic pressure (Pa), distal intracoronary pressure (Pd), and hyperemic Tmn were measured in triplicate during sustained hyperemia. FFR was calculated as the mean Pd – to - mean Pa ratio. CFR was calculated as the resting-to-hyperemic Tmn ratio and IMR was calculated as Pd*hyperemic Tmn. When the pressure sensor was pulled back in the guiding catheter, both pressures were checked to exclude any drift of the transducers. A final aortic-to-distal pressure difference within ± 2 mmHg was considered acceptable. Normalization and physiological assessment were repeated for larger pressure offsets. Patients were classified into the reference group (CFR ≥ 2.5), functional CMD group (CFR < 2.5; IMR < 25), or structural CMD group (CFR < 2.5; IMR ≥ 25) [17].

### Echocardiography

All patients benefited from a standard echocardiographic examination using a commercially available machine (Vivid E95, General Electric Medical Systems, Horten, Norway) and a 2.5 MHz transducer. Images were acquired in the left lateral decubitus position. Blood pressure was recorded using a digital blood pressure monitor with a brachial cuff. A complete M-mode and two-dimensional gray scale echocardiography, including the 3 standard apical views (4, 3 and 2 chambers) using high frame rates (> 60 frames/s), were performed for each patient. All echocardiographic data were synchronized to the electrocardiogram. Image analysis was independently performed by a single blinded observer unaware of coronary status. The following measurements were recorded according to the American Society of Echocardiography (ASE) and the European Association of Cardiovascular Imaging (EACVI) guidelines [18]: LV end-diastolic and end-systolic volumes and ejection fraction using Simpson’s biplane method from apical 2- and 4-chamber views, left atrial volume index using a biplane area-length formula at end-systole. LV diastolic function was evaluated with E/e’ ratio, derived from the pulsed wave tissue Doppler images (TDI) at the lateral mitral annulus. Tricuspid annular plane systolic excursion was evaluated by applying M-mode in the apical 4-chamber view and used as a measure of RV systolic function. Systolic pulmonary artery pressure was estimated as the sum of the peak tricuspid regurgitation and right atrium pressure derived from the inferior vena cava diameter and inspiratory collapse. Color Doppler and pulsed wave Doppler were used to explore for the presence of valvular regurgitation, while continuous wave Doppler was used to quantify valvular stenosis obstruction. Commercially available software (EchoPAC PC version 203; GE Medical Systems, Milwaukee, WI, United States) with a speckle-tracking technology was used to measure LASr. As recommended by the European Association of Cardiovascular Imaging and the American Society of Echocardiography task force for deformation imaging, strain was evaluated on a non-foreshortened apical 4-chamber view with zero reference set at end-diastole (i.e., R-R gating) [19]. The LA endocardial border was automatically drawn followed by manual adjustment if required.

### Statistics

Continuous variables are expressed as mean +/- SD for normally distributed variables and as median (25th–75th percentile) for non-normally distributed variables. Discontinuous variables were presented as percentages. Data comparisons between the two patient groups were carried out for continuous variables using a Student’s t-test for parametric series, or a non-parametric Mann-Whitney test in the case of non-normal distributions and using Chi 2 or Fischer test for discrete variables. Pearson’s correlation coefficient was used for bivariate correlation analysis. Univariate and multivariate logistic regression analyses were used to quantify the association between CMD and the independent variables using odds ratios (OR) and 95% confidence intervals (CIs). Factors clinically recognized risk factors were included in the multivariate analysis (age, high blood pressure, body weight Index, diabetes, smoker). ROC curve analysis was used to assess the ability of LASr to predict CMD. Coefficient of variation was used to determine the variablity of data. The intra-observer and inter-observer variabilities of LASr measurements were quantified by calculating the intra-class correlation coefficient (ICC). Inter-observer variability was assessed using a two-way randomized single-measure ICC analysis. Intra-observer variability was assessed using a one-way random two-measure ICC analysis. For all statistical tests, the alpha threshold was set at 5%. Statistical analysis and graphs were performed using SPSS 24.0 (IBM Software).

## Results

A total of 42 patients were included in the analysis, out of which 26 had CMD, while the remaining 16 were free of microcirculatory involvement. Clinical parameters were comparable between the two groups, with a majority of women in both. Population characteristics are summarized in Table 1. No patient presented parossistic or permanent atrial fibrillation.

**Table 1:** Population characteristics

Parameters	CMD	Control group	*p* value
	*n* = 26	*n* = 16	
Male Sex (% male)	11 (42)	5 [31]	0.4
Age, years (± SD)	62.9 ± 9.07	59.6 ± 11.7	0.3
Body weight Index (Kg/m2)	27.9 ± 4.99	25.8 ± 4.92	0.19
Smoker (n, %)	8 [30]	5 [31]	0.97
Diabetes (n, %)	13 (50)	4 [25]	0.10
Hyperlipidemia (n, %)	9 (56)	15 (58)	0.92
High blood pressure (n, %)	11 (42)	6 (38)	0.75
Coronary heredity (n, %)	2 [8]	0 (0)	0.51
Renal clearance, ml/mn/m2 (± SD)	81.9 ± 19.0	87.5 ± 18.5	0.35
Symptoms according to Diamond and Forrester			
Typical angina	11 (42)	0 (0)	0.003
Atypical angina	9 (35)	13 (81)	0.003
Non anginal	1 [4]	0 (0)	0.6
Dyspnea	18 (69)	3 [19]	0.001

### Coronary microcirculation dysfunction

The results are presented in Table 2. All patient in the CMD group presented with structural CMD.

**Table 2:** Invasive coronary physiology

	CMD (*n* = 26)	Control group (*n* = 16)	*p* value
Systolic blood pressure (mmHg)	125.3 ± 25.5	127.3 ± 11.5	0.77
Diastolic blood pressure (mmHg)	73.1 ± 8.51	70.5 ± 10.5	0.38
Heart rate (bpm)	68.5 ± 12,1	67.8 ± 9.27	0.88
Rate Pressure product	8522 ± 2226	8633 ± 1340	0.85
Baseline Tmn (s)	0.86 ± 0.33	0.55 ± 0.21	0.002
Hyperhemic Tmn (s)	0.50 ± 0.25	0.16 ± 0.06	0.001
Pa hyperhemic (mmHg)	84.0 ± 22.3	90.6 ± 14.2	0.03
Pd hyperhemic (mmHg)	77.1 ± 12.9	86.8 ± 9.39	0.03
FFR	0.91 ± 0.04	0.91 ± 0.03	0.63
CFR	1.78 ± 0.49	3.48 ± 1.03	0.0001
IMR (mmHg x s)	29.5 (28.2–43)	15 (10.7–17.7)	0.0001

FFR: fractional flow reserve; CFR: coronary flow reserve; IMR: index of microcirculatory resistance, Pa: aortic pressure; Pd: distal coronary pressure, Tmn: mean transit time at rest

### Echocardiographic analysis

**Fig. 2:**
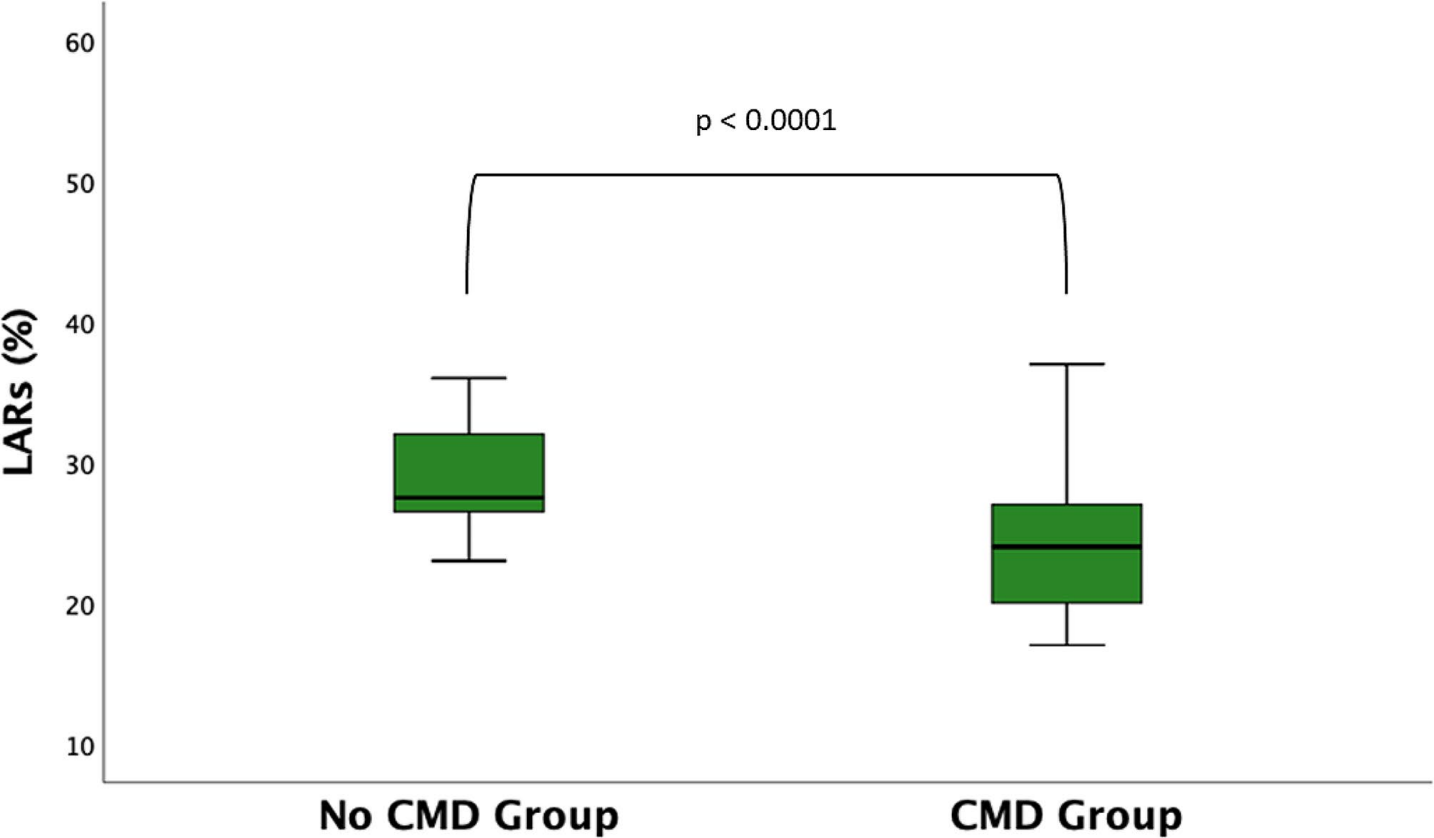
Comparison between CMD and No CMD groups. CMD: Coronary microvascular dysfunction; LASr: Left atrial strain reservoir

There were no significant differences between CMD-positive and negative patients on the basis of classic echocardiographic functional and structural parameters. LASr was significantly reduced in the CMD group in comparison with the control group (24.6% ± 6.1 vs. 30.3 ± 7.8%, *p* = 0.01) (Fig. 2). Echocardiographic characteristics are summarized in Tables 3, 4.

**Table 3:** Echocardiographic characteristics of patients

	CMD (*n* = 26)	Control group (*n* = 16)	*p* value
LVWT (cm)	0.86 ± 0.17	0.88 ± 0.15	0.67
LVMI (g/m2)	79.3 ± 18.6	73.2 ± 17.6	0.29
LVDI (ml/m2)	52.7 ± 13.9	50.1 ± 10.4	0.37
LVEF (%)	63.1 ± 6.67	63.0 ± 5.09	0.39
E (m/s)	7.25 ± 3.02	6.44 ± 1.33	0.32
A (m/s)	6.92 ± 2.81	7.8 ± 2.24	0.28
E/A	0.90 ± 0.29	0.86 ± 0.22	0.69
E’(cm/s)	10.5 ± 4.55	9.01 ± 3.81	0.25
E/E’	6.95 ± 2.95	6.58 ± 2.86	0.68
PAPS (mmHg)	24.6 ± 6.64	20.4 ± 4.58	0.06
TAPSE (mm)	23.6 ± 4.63	21.8 ± 3/03	0.16
Peak S Annular tricuspid (cm/s)	12.4 ± 2.42	12.0 ± 1.41	0.49
LAVI (ml/m2)	31.0 ± 12.8	28.0 ± 6.44	0.39
LASr (%)	**24.6 ± 6.14**	**30.3 ± 7.79**	**0.01**

LAVI: left atrial volume index, LASr: left atrial strain reservoir function, LVDI: LV dimensions at end-diastole index, LVEF: left ventricle ejection fraction, LVMI: left ventricular masse index, LVWT: left ventricular wall thickness, PAPS: pulmonary artery pressure systolic, TAPSE: Tricuspid annular plane systolic excursion

**Table 3:** Univariate and multivariate logistic regression analysis for coronary microvascular dysfunction

Variables	Univariate		Multivariate	
	OR (95% CI)	*P* value	OR (95% CI)	*P* value
Age	1.033 (0.970–1.101)	0.309		
Body weight Index	1.095 (0.954–1.257)	0.19		
High blood pressure	1.22 (0.341–4.381)	0.75		
Smoker	0.97 (0.255–3.75)	0.97		
Diabetes	3.00 (0.764–11.78)	0.15		
LASr	0.87 (0.78–0.98)	0.02	0.88 (0.78–0.99)	**0.04**

#### CMD and LASr

**Fig. 3:**
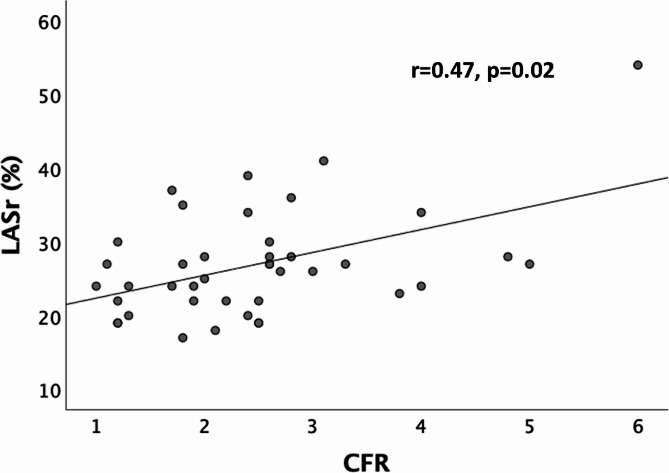
Correlation between LASr and CFR. CFR: Coronary flow reserve; LASr: Left atrial strain reservoir

**Fig. 4:**
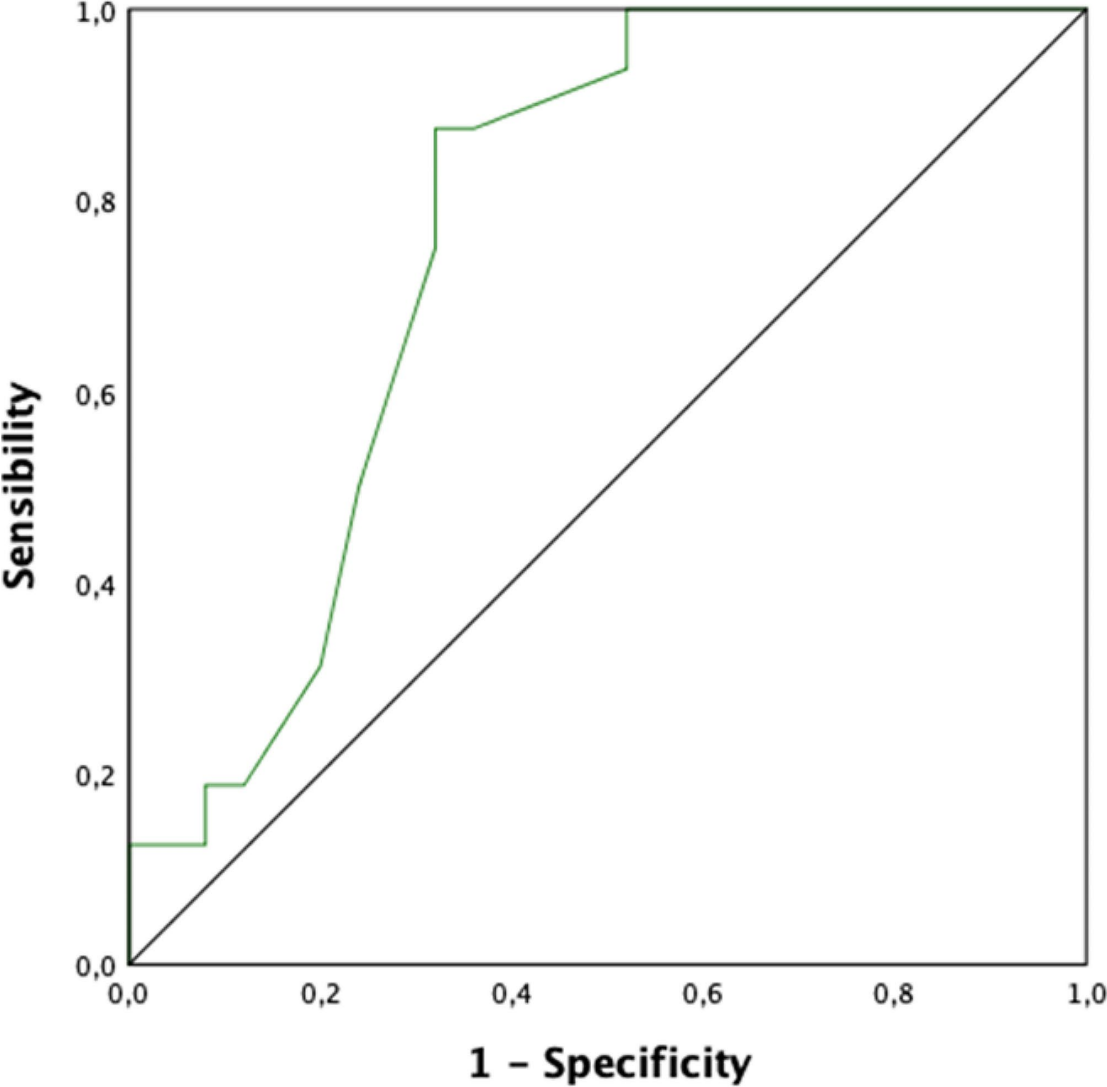
ROC curve for the assessment of the diagnostic performances of LASr for CMD

There was a moderate yet significant positive correlation between LASr and CFR (*r* = 0.47, *p*=0.002) (Fig. 3). With regards to diagnostic performance, the ROC curve presented in Fig. 4 indicated an area under the curve of 0.76 (0.62-0.90) (95% CI), corresponding to a parameter with relatively high discriminatory potential. The LASr cut-off value was 25.5%, enabling better classification of patients with or without CMD.

### Reproducibility

Intra- and inter-observer variability of LASr assessment was good. The results are summarized in Table 5.

**Table 4:** Assessment of inter- and intra-observer reproducibility

	Intra-observer reproducibility		Inter-observer reproducibility	
	Coefficient of variation %	ICC	Coefficient of variation %	ICC
LASr	26.6	0.92 (0.86–0.96)	27	0.82 (0.67–0.90)

## Discussion

The main result of this study was that patients with CMD had a significantly lower LASr than patients without CMD and a multivariate logistic regression analysis demonstrated that CMD was independently associated with LASr.

The strength of this study is that coronary microcirculation was assessed using a robust method validated by international consensus [3, 4, 20, 21]. We showed that LASr was the only TTE parameter to be significantly more altered in the CMD group than in the control group. Advantages of LASr over standard diastolic function parameters include independence of the acquisition from the measurement angle, low influence of loading conditions, and excellent inter- and intra-individual measurement reproducibility. Studies have shown thar LASr diagnostic performance was actually superior to that of other parameters. As demonstrated in the work by Reddy et al., LASr appeared to be the most discriminating parameter in the diagnosis of diastolic dysfunction in patients with HFpEF [22]. Compared with standard multiparametric measurements of diastolic function, LASr evaluation is rapid and easy for expert investigators. It provides information on a gradual, linear impairment of left atrial function [9, 11].

Patients with cardiovascular risk factors such as obesity, hypertension and diabetes exhibit low-grade systemic inflammation. These factors lead to lesions of the coronary endothelium and, consequently, to structural and functional abnormalities of the microcirculation [7, 23]. Structural CMD correspond to a reduction in vessel lumen due to hypertrophy of vascular smooth muscle cells, hypertrophy of myocyte cells, interstitial fibrosis, vasodilatory impairment, and/or hyperreactivity to vasoconstrictor stimuli. Functional CMD correspond to abnormal vasodilation at rest [24]. In our study, structural CMD was only observed. Interesting, no patient in this prospective cohort presented parossistic or permanent atrial fibrillation. This may be explained by the screening method, which targets INOCA patients with a systematic LV end-diastolic pressure measure in accordance of expert consensus [4]. All patients had normal LV end-diastolic pressure (≤ 10 mmHg). This information is important because it was not available in the other studies on the subject, and enables a mechanistic hypothesis to be made. In the case of INOCA, CMD is responsible for an early disorder of diastolic function, with a relaxation disorder but no increase in filling pressures. Relaxation is an active phenomenon, due, at cellular level, to the relaxation of fibers by release of actin and myosin bridges, which requires reuptake of intracellular Calcium into the sarcoplasmic reticulum by the Sarcoendoplasmic Reticulum Calcium ATPase (SERCA) pump. This is an energy-consuming process, which explains the early diastolic disturbances seen in ischemia. For certain teams, INOCA represents a pre-HFpEF state. An increase over time in structural anomalies of the coronary microcirculation leads to ventricular filling disorders due to impaired myocardial compliance which can in turn lead to the development of HFpEF [7, 25]. Several data show correlation between CMD and HFpEF [26]. As shown by the PROMIS-HFpEF study, up to 75% of HFpEF patients have CMD [5]. Anatomopathological studies revealed that patients with HFpEF had decreased capillary density and increased fibrosis compared to subjects without heart disease [27]. A recently published meta-analysis concluded that the diagnostic and prognostic values of LASr were convincing in HFpEF [28]. European cardiac imaging guidelines now suggest the inclusion of LASr results in the new algorithm designed for the estimation of left ventricular filling pressures [29].

Our results contribute to demonstrating the link between left ventricular diastolic abnormalities and CMD in NOCA patients without HFpEF. In the literature, the link between reduced LASr and CMD has already been explored. Keulards et al. concluded that there was a correlation between increased coronary microvascular resistance and impaired LASr. A relationship was also observed between the intensity of microcirculatory damage and altered strain [30]. The authors used continuous thermodilution, whereas bolus thermodilution was used in our study. The former method has been shown to be less prone to measurement variations. However, it requires the use of a dedicated microcatheter (Rayflow®, Hexacath, France), making the procedure more time-consuming, and for the time being, cut off decisions and international expert recommendations recommend the use of bolus thermodilution [4]. Similarly, Konerman et al. found a correlation between altered LASr and CMD as assessed by positron emission tomography. The results also showed that left ventricular systolic parameters such as global longitudinal strain (GLS) did not discriminate between patients with and without CMD [31]. In addition to confirming such data, our results indicate that the use of bolus thermodilution is suitable to evidence the relationship between LASr and CMD. If the present results will be confirmed by several larger studies, LAsr could be used for two purposes in the CMD field. First, LASr could help to classify NOCA patients at high risk of CMD. Second, LASr as an easily measurable, noninvasive and reproducible parameter may ultimately enable the therapeutic monitoring of CMD.

### Limitations

This study was monocentric and included a relatively small cohort of patients. The results therefore require further validation on a larger sample. Unfortunately for the LV GLS, the number of non-analyzable segments was too large to provide a robust data set, and we preferred not to analyze these data. In addition, it is important to mention that the echocardiographic study was performed at rest. It should also be noted that strain analysis was carried out on a single equipment while strain values may vary according to the manufacturer.

## Conclusion

LASr represent an easily measurable, reliable, and reproducible indicator of diastolic function. LASr was significantly impaired in patients with CMD, indicating diastolic function impairment. LASr could be integrated into the screening, diagnosis, and follow-up of CMD patients.

## Data Availability

The data that support the findings of this study are available on request from the corresponding author.

## Abbreviations

BMI: Body Mass Index
CFR: Coronary Flow Reserve
COVADIS: Coronary Vasomotor Disorders International Study
CMD: Coronary Microvascular Dysfunction
ESC: European society of Cardiology
FFR: Fractional Flow Reserve
GFR: Glomerular Filtration Rate
HFpEF: Heart Failure with preserves ejection fraction
HTA: Hypertension
IMR: Index of microcirculatory resistance
LASr: Left atrial strain reservoir
LDLc: Low-Density lipoprotein cholesterol
LA: Left atrium LAVI left atrial volume index
LVEF: Left ventricular ejection fraction
LVWT: left ventricular wall thickness
LVMI: left ventricular masse index
LV: dimensions at end-diastole index (LVDI)
PAPs: Pulmonary arterial systolic pressure
RPP: Rate Pressure Product
SBP: Simpson Biplan
TAPSE: Tricuspid Annular Plane Systolic Excursion
TTE: Trans-thoracic echocardiography
